# Effect of COVID-19 on Dental Education: A Review

**DOI:** 10.7759/cureus.24455

**Published:** 2022-04-25

**Authors:** Lakshmi Trivandrum Anandapadmanabhan, Pratibha Ramani, Ramya Ramadoss, Suganya Panneerselvam, Sandhya Sundar

**Affiliations:** 1 Oral Pathology, Saveetha Dental College and Hospitals, Chennai, IND

**Keywords:** effect, impact, dentistry, dental education, covid-19

## Abstract

Corona Virus Disease (COVID-19) has become a pandemic and a real threat for those working in healthcare. It has affected dental professionals in education, research, and practice. This article intended to review the impact and brunt of COVID-19 outbreaks on dental education and research country wise and how it affected the three learning domains - cognitive domain, psychomotor domain and affective domain. A review was designed to identify the impact of the COVID-19 epidemic on dental education. A literature search was conducted using PubMed, Scopus and Google scholar databases. Studies in which teaching methods by virtual means were described and how it affected dental education during the pandemic all over the world were included. The search terms selected to search for literature were dental education, COVID-19, dental schools and dentistry. COVID-19 has caused a significant change in overall dental education in all the countries. It has affected dental education both negatively and positively. The pandemic caused disruption in learning leading to psychological distress. Even though many students preferred web-based learning, majority of them considered learning through online mode as a challenge. COVID-19 pandemic has enforced all the dental schools to modify their traditional way of teaching to an alternate mode of teaching to adapt to this current situation of the pandemic all over the world. It caused an everlasting impact on dental education, research along with practice. The dental institutes must be well prepared to face this pandemic by investing in educational software so that it leads to the evolution and advancement in the fields of virtual mode of teaching, in research and in preclinical training.

## Introduction and background

**Figure 1:**
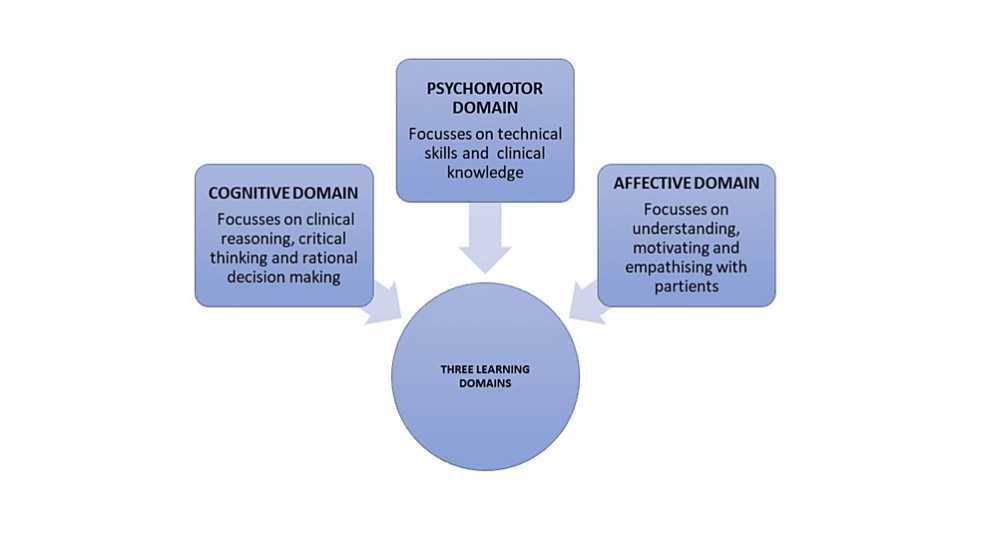
Three learning domains in the dental curriculum

The Coronavirus Disease (COVID-19) is a novel disease that evolved in Wuhan, China in 2019 [1]. It has disseminated all over the world and has become a pandemic [2,3]. It caused a real challenge to health care providers all over the world and has undoubtedly affected dental professionals not only in practice but also in research and dental education. The quarantine and lockdown linked with the COVID-19 pandemic have compelled universities and dental institutions to put their clinical training on hold and to switch to online programs for distance learning. All dental colleges and institutions are ensuring that dental students satisfy the Dental Council's outcomes, and they are doing all possible to provide the greatest education to students through various ways. The three learning domains in dental curriculum include cognitive domain, psychomotor domain and affective domain (Figure 1). The COVID-19 pandemic has affected all three domains in the dental curriculum. For the past few months, learning through virtual means has become the only option for continuing education all over the world [4]. This article intended to review the impact and brunt of COVID-19 outbreaks on dental education and research country wise and how they impacted the three learning domains.

## Review

Impact of COVID-19 on dental education in the USA

The influence of COVID-19 on dental education in the United States was investigated by Iyer et al. [5]. They discussed the challenges faced by dental school administration and in teaching and learning. According to them, the major challenge faced by dental school administration is to safeguard the health of students, faculty, and patients and also to make sure that students have no hindrance in the continuity of dental education. They also noticed that most dental schools in the United States have ceased clinical activity except in emergencies, and that some preclinical simulation laboratories use social distancing. While some institutions regard mannequin simulation as a challenging technique of instruction, virtual reality systems and haptic technologies are not portable [5].

In some academic disciplines where in-person curricular requirements are required for effective training, the disease has harmed and disordered the learning process. This pandemic had an impact not just on student learning but also on the economy. Because there was reduced patient flow during and after the epidemic, dental schools' income was reduced. There will be an increasing financial burden placed on dental students, since some of them may be in debt, and this may result in a higher student drop-out rate in dental schools, leading to a reduction in the future dental healthcare workforce and creating a great impact on the future scope of dentistry. In addition to this, students who apply for advanced dental programs face an economic burden and there will be a delay in processing their applications [1]. In another study, the impact of COVID-19 on dental education and dental students was analyzed and they found out that most of the dental students experienced greater levels of stress and also felt that their clinical education has suffered a lot [6].

Only nine states presently do not have regulations limiting dentistry practice, according to the American Dental Association, which has urged dental practitioners to provide emergency care for patients [7]. The COVID-19 pandemic has been linked to poor mental health among dentistry students, according to studies. The majority of them agreed that this pandemic caused generalized anxiety disorder and depression. Due to the rigor and severity of dental school, the COVID-19 epidemic may exacerbate the consequences of poor mental health, to which dental trainees are vulnerable. They also encounter increased skepticism about their academic and clinical training, as well as their work and income prospects in the future. The virtual mode of education prohibits socialization and personal interaction between students and the teacher. Many of them had dropout intentions. This can be minimized by psychological counseling services, providing comprehensive wellness programs to all trainees, and engaging them with extracurricular social activities [8].

Impact of COVID-19 on dental education in Jordan

A survey was conducted among dental students by Hattar et al. and they investigated the impact of COVID-19 quarantine on the willingness of dental graduates, and they explored the adequacy and constraints of online education from the perspective of students [2]. They discovered that while dental students acknowledged the online method of dental education, they did not consider it a substitute for one-on-one clinical experience in their study. They also concluded that students had some apprehensions concerning self-reliant dental practice following their graduation [2,9].

Impact of COVID-19 on dental education in Brazil

Machado et al. discussed current and future perspectives of the pandemic's influence on dental education. They emphasized that platforms like Google educational tools, Skype, Facebook, Instagram, Youtube, WhatsApp, Telegram, LinkedIn, Pinterest, Zoom, Jitsi, Microsoft Teams, and Webex are used extensively worldwide for learning in this situation. Other platforms like Google Meet and Microsoft Teams are also used. Intriguing research found that when doing problem-based learning using Webex, students were distracted by phone calls and web browsing [10].

Dental schools are unable to fully implement web-based instruction in dental practice, thus some short considerations should be taken. Activities of dental schools should be resumed in classrooms and clinics and this has to depend upon the pandemic in each country. Some dental schools have resumed courses or are preparing to reopen as a result of government decisions, and it is highly advised that dental education be sorted out in light of the "new normal" in dentistry [11].

Impact of COVID-19 on dental education in Saudi Arabia

All colleges worldwide have been facing devastating pandemic outbreaks and lockdowns creating difficulty in clinical learning by dental students. In addition to this, no treatment for patients in clinics also poses a challenge to dental education. Other issues include financial loss, economic instability, interruption of research programmes and funds, as well as the cancellation of conferences and convocations [12].

The virtual education system has replaced in-person face-to-face learning systems all over the world. Students enrolling in massive open online courses were shown to be extremely motivated to learn using numerous creative instructional tools such as video and interactive animations, according to research. Flip classrooms are another learning approach in which students are initially introduced to courses through online classes and then have a face-to-face session for active learning [12].

Many colleges have developed procedures that provide faculty members more time to make up for their lower research productivity before their promotion is necessary [13].

Impact of COVID-19 on dental education in Pakistan

Manikins, virtual reality or augmented reality-based stimulation devices, and haptic technologies, according to Haroon et al., have been particularly effective for dentistry education [14].

Impact of COVID-19 on dental education in India

During the COVID-19 pandemic in India, Shrivastava et al. performed a cross-sectional virtual survey to investigate the effectiveness of online dental education systems among undergraduate dentistry students. They came to the conclusion that pandemics have a negative impact on dentistry education. Dental students' physical and psychological well-being was harmed, particularly among males. The quality of online education should be improved and educational criteria should be met. The majority of dentistry students are worried about their studies and approaching university exams. This was due to a lack of engagement between students and interested faculty members, as well as ambiguity over examination dates [15]. Even after the COVID-19 pandemic, dentists face a variety of issues, highlighting the need for significant reforms in dentistry, both in practice and in education.

To maintain proper distance learning etiquette, a suitable plan must be devised and it must be flexible enough to react to any complaints that may arise. The perspective and collaboration of both students and professors are critical to the virtual mode of teaching's success. The ability of students to acquire wisdom online, as well as the qualities of online information, all play important roles in the success of online education. The ease with which students transfer from traditional to virtual learning is influenced by a variety of cultural, economic, and political factors. Both synchronous teaching with peer interactions and asynchronous teaching by professors must be combined for a better educational experience. They also mentioned that the flipped classroom and problem-based learning can be utilized to assess how well students understand what they learned in the prior class. Dental educators and administrators should examine the security of their students, patients, and other staff, as well as the continuity of instruction, before using computer-based learning methods. The lack of direct patient care instruction is a major deterrent for students, and the key issue for instructors is to keep students engaged and inspired during online classes [16].

Dental education is a challenging field that necessitates much training. For successful graduations, final assessments and examinations have been changed and adapted [17].

Impact of COVID-19 on dental education in China

According to Guo et al., the COVID-19 epidemic has also influenced people's utilization of emergency dental services and they found out that there was a decline in utilization of emergency services at the onset of the epidemic and it firmly influenced emergency dental services in China [18].

Even though several authors have highlighted the impact of COVID-19 in dental education and practice, only Hung et al. contributed recommendations for dental education [6]. According to them, when COVID-19 infection is suspected, instructions on infection prevention and control are recommended. Dentists should take stringent personal protection measures and avert procedures that involve aerosols or droplets. When it comes to dental education, open communication between dental students, clinical teachers, and administrative personnel strengthens mutual trust and makes it easier to provide enough assistance. They advocate using online lectures, case studies, and problem-based learning tutorials for dentistry education during an epidemic, which must be endorsed to avoid unnecessary aggregation of people and the associated risk of infection. Students can now observe and examine lectures whenever and wherever they want thanks to existing smart gadgets and applications. All the students should be reassured to engage in individual training and update themselves on the latest academic developments. Another important recommendation suggested by them is that dental students can be afflicted by panic and anxiety, stress and tension and all dental schools should have a plan in place to help students who require it.

Impact of COVID-19 on dental education in Italy

After a month of distance education, Bennardo et al. came out with the following findings: Online examinations are not an excellent approach of evaluating students in health education and they added that it would be possible to analyze students' skills only theoretically through this means. Many students and instructors have appreciated e-learning, particularly in terms of student-teacher collaboration. They also stated that remote activities cannot completely replace clinical coaching. As a result, these issues should be addressed in the second semester. Free educational software, such as G Suite for Education and Microsoft Office 365 Education, can be used by universities with limited resources. This allows students to communicate with one another using a variety of programmes for meetings and file sharing [19].

Impact of COVID-19 on dental education in France

COVID-19 had impacted on dental education a lot that tutoring of fine motor skills was disrupted because of social distancing. Many are reviewing whether preclinical training can be done from home. Development and investment of virtual and haptic technology have various drawbacks like using them is not a common method of evaluating students, high cost, and not easily movable. So, they suggested the use of simple, handy manikins, which are economical, weightless, and compact and they can be adhered precisely to a desk at home. Furthermore, a contra-angle portable micromotor will assist students in completing their practical work. This also allows students to gain additional experience before entering clinics. During online lessons, portable equipment also allows students to practice and collaborate in real time with lecturers. Visual work control that is not synchronous should be anticipated [20].

The online mode of education has various advantages. This includes allowing students to study at their own speed in a topic that interests them the most in a location that is convenient for them, time-saving, facilitating participation from students who are from different range of learning abilities, improving the working hours of educators, no hassle of faculty shortage, facilitates collaborative learning and change from the teacher-centric mode of education, which is passive, to a learner-centric model of education which is more interactive. E-learning also focuses on the learner's needs, develops a learning environment free of time and logistics constraints, and helps students to make the most of their time and resources. The virtual form of education has a number of drawbacks, including a lack of socialization and human connection between students and teachers, internet connectivity concerns, and the requirement that every student has for the appropriate equipment to continue their education without interruption. According to research, the online method of teaching necessitates more time and resources from educational institutions to produce online study materials and technological assistance [21,22].

A recent review done by Gaudin et al. in France emphasized that e-learning, video conferences, and webinars should be encouraged for continuing education. Emails and phone conversations were utilized to communicate with students, while Moodle platforms were employed to assist curriculum delivery and material. The Moodle platform was utilized by the majority of dentistry schools in France to administer online examinations. This resulted in the least amount of inconvenience for the learners. From the traditional circumstance to the current COVID-19 situation, online tests were administered using this means. For theoretical and practical information, the percentage of missing content was estimated to be between 10% and 15%. They also discovered that the COVID-19 epidemic has had an influence on the academic calendar. They also stated that students who are less productive and efficient are to be expected. There is a 20% decrease in patient volume and income as compared to previous years [22].

Impact of COVID-19 on dental education in Hong Kong

In Hong Kong, Wong et al. investigated the impact of COVID-19 on educational interventions as well as endodontic practice [23]. Clinical, practical, and interactive learning have all been impacted in dental education in Hong Kong. Several modifications were done in the academic timeline like postponement of Bachelor of Dental Surgery (BDS) examinations and use of online method of conducting examinations, use of problem-based learning and video conferencing platforms. There have been no reports of COVID-19 transmission between patients and dentists in Hong Kong. For endodontic treatment, strict infection control measures were followed and rubber dam isolation for moisture control was done for ensuring an aseptic working field.

Various modes of online learning, their advantages, and disadvantages

Google Meet allows for live activities with up to 250 people, as well as the ability of sharing the presenter's screen and numerous pedagogic actions. Google Drive or Google Classroom may be used to record and save activities. Various changes to the educational system are required to learn preclinical and clinical e-learning activities. It has been discovered that employing virtual slides with the entire slide picture for oral pathology instruction is beneficial. It outperforms the standard method of utilizing conventional microscopy. Several technologies scan the glass slide to create high-resolution digital slides or whole-slide image. The whole image of the slide can be saved on the institution's drives or servers.

Evaluation of final assessment

Undergraduate and postgraduate students lost many months of clinical practice and they were unable to complete their entire programmes due to a lack of resources. It was difficult to measure students' knowledge and skills, and the repercussions may be seen in the coming year [22].

Group discussions, clinically important case presentations, case-based and team-based learning, and timed quizzes can all be used to assess the impact of online training. Final certification includes an objective structured clinical examination (OSCE) as well as peer-reviewed assessments of their clinical work completed previously via webinars or teleconferences in the presence of a team of supervisors. Various tests, as well as graded projects and exams, will aid in breaking down the course into manageable parts, allowing the student to develop.

Effect of COVID-19 on patient management

Dental emergency patient management includes the management of severe pain, recurring infection and trauma. All dentists should limit their dental care to exclusively emergency cases during the COVID period. To reduce the danger of virus transmission, personal protective equipment should be used. To decrease contamination during dental procedures, rubber dam isolation might be done [21].

Dental students believe that mentoring is very important in patient management, yet they still have reservations about practicing independently. This could be linked to the amount of practice they got in their last years. Exposing students to community dental care, which has proven to be a helpful supplemental form of clinical experience, communication skills, and self-confidence, is one strategy to overcome this concern [2]. Only 11% of undergraduate students were invited to participate in non-clinical activities in dental facilities, where senior personnel did 96 percent of clinical work with 30 percent participation of postgraduate students [12].

Effect of COVID-19 on dental research

The cessation of most laboratory-based dental research projects and postgraduate student research projects was unavoidable due to mandatory government and institutional laws restricting research operations. Some are concentrating their efforts on conducting online surveys and literature studies.

Students in Ph.D. programmes and speciality training do not have the opportunity to attend symposiums and conferences. As a result, they miss out on the opportunity to exchange ideas and communicate their findings to their superiors and peers. Students should be encouraged to write and publish research works making use of this time with the help of their mentors [3,21].

Many institutions have developed procedures that provide faculty members more time to compensate for lower research productivity before they are promoted [13].

Recommendations to improve dental education

**Figure 2:**
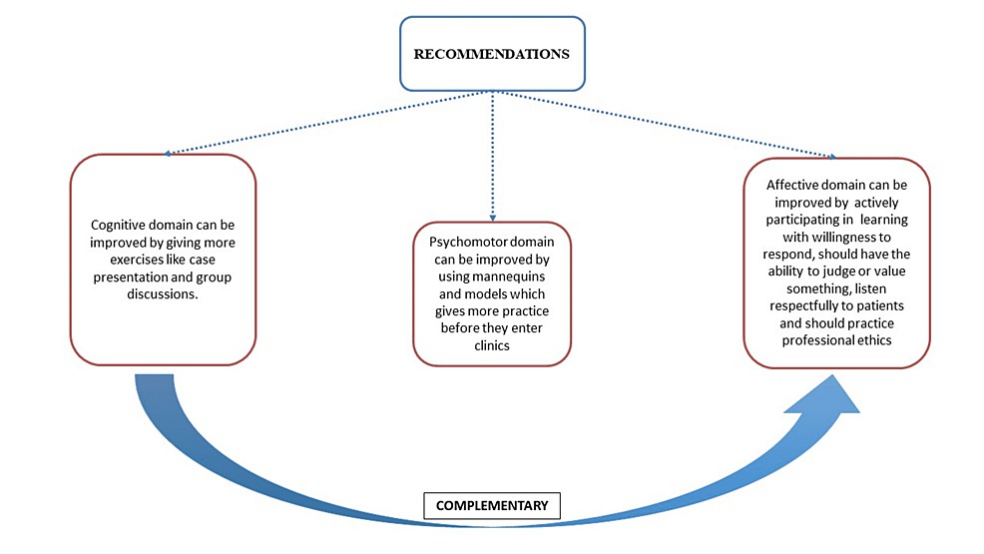
Recommendations to improve three learning domains in dentistry during the pandemic

Various steps have to be taken to improve all three domains of the dental curriculum in this pandemic. Cognitive domain can be improved by giving more exercises on clinical reasoning through group discussions and case presentations. This also enhances critical thinking and rational decision-making. Psychomotor domain can be improved by using various modern training resources like mannequins and models. Dental students should actively participate in learning with a willingness to respond, should have the ability to judge or value something, listen respectfully to patients and should practice professional ethics. This helps in improving the affective domain (Figure 2). Affective and cognitive domains are complementary to each other [24].

Simulating clinical scenarios to improve students' decision-making and diagnosing abilities is what virtual patient-based learning is all about. Dental students' learning has increased as a result of a web-based virtual patient-based training research on herpes simplex infection and recurrent aphthous stomatitis. Case-based discussions may also be possible on these learning platforms. In light of this new reality, we should reassess our curriculum and how we give lessons and lectures [21].

In many dental institutions, preclinical simulation activities were maintained on campus, and social distancing measures were developed and implemented. To allow for more hands-on exposure, some universities changed their assessment plans or extended program finish dates [13]. Various studies were done to know the impact of COVID-19 on dental education and research in various countries (Table 1).

**Table 1:** Research done on the influence of the COVID-19 epidemic on dental education in several nations

Country	Authors and year	Study design	Number of participants	Findings
Jordan	Hattar et al., 2021 [2]	Cross-sectional survey	432	Students partially appreciated e-learning, not a substitute for face-to-face clinical practice
Many countries	Ammar et al. [4]	Cross-sectional online survey	1862 academics from 28 countries	The pandemic caused a considerable psychological impact on dental academics
USA	Hung et al. [6]	Cross-sectional online survey	145	Students experience increased levels of stress due to pandemic and felt their clinical education had suffered.
	Chi et al. [8]	Cross-sectional online survey	355	COVID-19 has exacerbated the prevalence and consequences of poor mental health among dental students
India	Shrivastava et al. [15]	Cross-sectional virtual survey	533	COVID-19 adversely affected dental education. Also affected the physical and psychological well-being of students
	Khan and Hashmi [17]	Descriptive online survey	928	Dental teaching and training very much disturbed during the pandemic
China	Guo et al. [18]	Retrospective analysis	2537	Pandemic had a strong influence on the utilization of emergency dental services

## Conclusions

Coronavirus disease is a type of viral pneumonia that is caused by SARS-CoV. It has affected people all over the world including healthcare workers. It affected dental professionals in education, research, and practice. The COVID-19 pandemic has caused all dentistry schools to change their standard teaching methods to an alternate manner of instruction in order to respond to the present pandemic crisis throughout the world. It caused many complications in the field of dentistry causing a long-term impact on dental education, research as well as practice in both developed and developing countries. It also affected the three learning domains - cognitive, psychomotor and affective domains. Dental educators also have adapted to this new method of digital teaching, thus affecting dental education in both negative and positive manner. The dental schools must be well prepared to face this pandemic by investing in educational software so that it leads to the evolution and advancement in the fields of the virtual mode of teaching, in research, and preclinical training.
